# Lifestyle Changes among Polish University Students during the COVID-19 Pandemic

**DOI:** 10.3390/ijerph18189571

**Published:** 2021-09-11

**Authors:** Karolina Fila-Witecka, Adrianna Senczyszyn, Agata Kołodziejczyk, Marta Ciułkowicz, Julian Maciaszek, Błażej Misiak, Dorota Szcześniak, Joanna Rymaszewska

**Affiliations:** Department and Clinic of Psychiatry, Wrocław Medical University, 50-367 Wrocław, Poland; adrianna.senczyszyn@student.umed.wroc.pl (A.S.); agata.kolodziejczyk@student.umed.wroc.pl (A.K.); marta.ciulkowicz@student.umed.wroc.pl (M.C.); julian.maciaszek@umed.wroc.pl (J.M.); blazej.misiak@umed.wroc.pl (B.M.); dorota.szczesniak@umed.wroc.pl (D.S.); joanna.rymaszewska@umed.wroc.pl (J.R.)

**Keywords:** mental health, online survey, COVID-19, coronavirus, lifestyle, university student, life skills

## Abstract

Students worldwide have been impacted by nationwide safety closures due to the COVID-19 pandemic, creating an environment with loss of interaction with colleagues, social isolation, boredom, and economic uncertainty. Since university students were considered uniquely vulnerable to mental health problems even before the pandemic, this study aimed to investigate lifestyle and behavioral changes experienced by this population due to the epidemiological situation and their effect on their mental health. Data were collected via an online survey conducted among university students across Poland. The survey addressed recent lifestyle changes that were a result of the pandemic as well as psychological distress, symptoms of insomnia and symptoms of post-traumatic stress. The results indicate that protective factors include maintaining a daily routine, staying physically active, following a usual eating pattern and taking care of sleep hygiene. Changes in behavior contributing to poorer mental health included giving up a daily routine, neglecting meals, tidiness, hygiene as well as social relationships, changes in food intake, sleeping schedule, a decrease in physical activity and the onset of sexual dysfunctions. A history of psychiatric treatment and an increase in self-harm as well as an increase in alcohol and tobacco consumption were also found to be associated with psychological distress. Experienced lifestyle and behavioral changes and their impact on mental health were apparent throughout the obtained data, highlighting the need for psychological support in the studied population. Based on the results we were able to establish a list of protective and risk factors influencing the everyday life and psychological wellbeing of students amidst the COVID-19 pandemic, which could also be translated into life skills.

## 1. Introduction

For more than a year, the entire globe has been affected by the coronavirus disease 2019 (COVID-19), caused by severe acute respiratory syndrome coronavirus 2 (SARS-CoV-2), which was first detected in December 2019 in the city of Wuhan, China. Currently, this pandemic has infected more than 177 million people in 222 countries around the world and has resulted in a devastating threat to human society in terms of health, economy, interpersonal relations, and daily habits. While public health recommendations (i.e., stay-at-home orders, closures of basic facilities and services) are necessary to protect public health, they may, however, dramatically influence individuals’ wellbeing and lifestyle-related behaviors.

As the spread of the epidemic continues, strict isolation measures and delays in starting schools, colleges, and universities occur across the globe. In Poland, since the 25th of March (with a short period break in September), all schools, colleges, and universities indefinitely suspended their attendance, and, where possible, activated online learning as an alternative [1]. According to UNESCO, over 87% of the students worldwide [2] have been impacted by similar nationwide safety closures. Due to this situation, students are prone to experience the loss of interaction with class colleagues, social isolation, boredom, and highly probable loss of part-time jobs in the world of the global recession caused by the COVID-19 crisis [3,4].

Even before COVID-19, it was clear that university students are uniquely vulnerable to mental health problems [5]. Being under a lot of pressure to perform academically, uncertainty about the future, relationship difficulties and feeling of loneliness are among causes which make them prone to develop unfavorable mental health outcomes [6]. The switch to online learning, isolation, fear of infection, and financial difficulties are very likely to exacerbate the already existing problems. For instance, in the Turkish study on perceived stress during the COVID-19 outbreak, more than half of the students of the study sample met the diagnostic criteria of generalized anxiety disorder (52%) and depression (63%) [7]. In the Spanish study on anxiety, depression and stress in the time of the COVID-19 outbreak, moderate to extremely severe scores of anxiety, depression, and stress were reported by 21.34%, 34.19% and 28.14% of the respondents (*n* = 2530), respectively [8]. Swiss longitudinal data collected since 2018 show that students’ levels of stress, anxiety, loneliness, and depressive symptoms got worse, compared to measures before COVID-19, and that main stressor for them was no longer a fear of missing out on social life, but worries about the ones they love [9].

Furthermore, in the time of economic shutdowns, quarantines and curfews also dangerous health behaviors, such as self-harm, eating disorders, elevated tobacco, drugs, and alcohol consumption, are more likely to be initiated [10]. Indeed, Canadian and Spanish studies showed that students’ high alcohol and tobacco consumption were significantly compounded during the pandemic [11,12]. Additionally, the confinement to the home may cause the interruption of the regular daily routine and a decrease in physical activity. In fact, it was found that the current quarantine negatively affected the practice of physical exercise by the student population [13]. Additionally, an international study undertaken by 35 research organizations from Europe, North-Africa, and Western Asia pointed out an increase in daily sitting time from 5 to 8 h per day during pandemic restrictions [14]. Moreover, constant mass-media reports about COVID-19 death rates lead to distress [15], and people under stress are more likely to eat more and choose less healthy ‘comfort foods’, mainly rich in simple carbohydrates [16,17], smoke more cigarettes [18], and more frequently reach for alcohol [19,20]. Furthermore, during the lockdown, there is a substantial risk of increasing substance use [21]. Interestingly, the pandemic has also influenced students’ sex lives, as most of them reported a decrease in sexual activity, yet use of dating apps remained unchanged [22]. Finally, the pandemic situation and lockdowns have also influenced students’ sleeping patterns—although they tend to spend more time in bed, overall sleep quality and insomnia symptoms have worsened [23,24,25]. All of these may negatively impact the mental health of students, who are initially threatened by addictions and mental crises.

While the majority of recent studies concentrated on the psychological impact of the COVID-19 pandemic on the general population or medical personnel, there is a need for more data about the student populations, as the pandemic has changed entirely their form of studying and interacting with other people. Thus, these are two of the most important areas of a student’s life.

## 2. Materials and Methods

### 2.1. Study Design

The study was conducted as a cross-sectional observational study. Data were collected via Computer Assisted Web Interviewing (CAWI) between the 12th of May and the 30th of June 2020. The survey was aimed at university students across Poland, and the link to the online form was distributed with the help of social media, various university websites and social media accounts as well as institutional e-mails among various universities across Poland. Eligibility criteria included university student status of any kind (including PhD students) at the time directly preceding data collection. The survey was anonymous and informed consent was obtained from all subjects involved in the study. Furthermore, to manage negative feelings related to the study the students had an opportunity to contact the psychiatry clinic of the Wroclaw Medical University via e-mail and to receive free psychological counselling, following the study. A number of participants took this opportunity to seek professional mental health advice and start mental health treatment. The study was conducted according to the guidelines of the Declaration of Helsinki and study protocol was approved by the Ethics Committee at the Wroclaw Medical University in Poland (no. 309/2020).

### 2.2. Participants

The studied group included a total number of 980 participants, within which 247 were male (25%) and 733 were female (75%). The mean age of the respondents was 22.24 ± 2.46 years. All of the participants were university students, the majority in their 1st or 2nd year (*n* = 447, 46%), which means their entire university experience was largely influenced by the pandemic and the changes it brought to educational environments. At the time the survey was conducted, COVID-19 exposure in the studied population was still at a low level. A detailed description of the demographic characteristics of the study group can be found in Table 1.

### 2.3. Measures

The online survey consisted of three sections. The first one aimed to collect sociodemographic information, including gender, age, residence, employment status, income source, relationship status, etc. The second section of the survey addressed recent lifestyle changes that were a result of the pandemic (i.e., a change in social activities, everyday routine, physical activity, relationships, eating habits, substance use, etc.). Sample items included “*were you able to keep your everyday routine during the pandemic”, “during the pandemic, have you noticed you neglect your [hygiene, meals, interests] more than usual”, “during the pandemic has the average time you spend on [sleep/physical activity] changed*”*?* The internal consistency of the questionnaire was calculated using Cronbach’s alpha and deemed satisfactory at α = 0.701. The last section of the survey included three questionnaires aimed to assess psychological distress (General Health Questionnaire, GHQ-28), symptoms of post-traumatic stress (Impact of Events Scale Revised, IES-R), as well as symptoms of insomnia (Insomnia Severity Index, ISI). The internal consistency of the used measurements in the studied sample was calculated using Cronbach’s alpha and presented as follows: GHQ-28 α = 0.941, IES-R α = 0.863, ISI α = 0.863. 

The General Health Questionnaire (GHQ-28) [26] is a 28-item, self-administered screening questionnaire, that allows to detect temporary or long-term changes in mental health that occurred in the studied population, as a result of current problems, a change in life circumstances or environmental event. It consists of four subscales: somatic symptoms, anxiety and insomnia, social dysfunction and severe depression. The items are rated on a 4-point Likert scale, from 0 (not at all) to 3 (much more than usual). The maximum score is 84 and is typically interpreted as higher scores indicating a higher possibility of psychopathological symptoms. For the purpose of this study the cut-off score indicating psychological distress was established at 24 points.

The Impact of Events Scale Revised (IES-R) [27] is a self-administered questionnaire with 22 items rated on a 5-point Likert scale. It describes the subjective level of stress related to a traumatic event. The questionnaire consists of three dimensions that relate to post-traumatic stress disorder symptoms, i.e., intrusions, arousal and avoidance.

Insomnia Severity Index [28] is a brief screening instrument to assess sleep problems and insomnia severity. It consists of seven items rated on a 5-point Likert scale concerning sleep onset, maintenance and problems awakening, dissatisfaction with sleep, interference of insomnia with functioning, and distress due to sleep problems.

### 2.4. Data Analysis

All of the received questionnaires that were complete and fulfilled the inclusion criteria (university student status) were included in the statistical analysis. Descriptive statistics (mean and standard deviation or counts and percentages) were used to calculate variables (demographic and clinical) and GHQ, ISI and IES-R scores. Differences in GHQ, ISI or IES-R between questionnaires’ responses were assessed using the Mann–Whitney test and Kruskal–Wallis test with the Holm correction for multiple comparisons. Additionally, effect sizes r (for Mann–Whitney test) or eta squared (for Kruskal–Wallis) were reported. To assess the independent effect of different questionnaires’ responses on GHQ, ISI and IES-R, multivariate linear regression was performed. The model was chosen on a theoretical basis and controlled by the AIC coefficient to assess for any significant confounding variables. The results presented below include the whole model. All analyses were performed in R for Windows, version 4.0.5 (R Foundation for Statistical Computing, Vienna, Austria). *p* < 0.05 was selected as the significance threshold.

## 3. Results

### 3.1. Main Findings

#### 3.1.1. Maintaining a Daily Routine and Productivity

Although most of the respondents agree that a daily routine is an important factor influencing the way they feel (*n* = 761, 78%), only a little over a third of the them were able to maintain it during the pandemic (*n* = 31, 7%). Moreover, the results indicate that individuals able to maintain their day to day routine scored significantly lower on psychopathological distress, as indicated by the GHQ scores. Maintaining a daily routine similarly significantly affected symptoms of insomnia and PTSD, where the better the routine was preserved, the lower the ISI and IES-R scores.

Most respondents (*n* = 760, 78%) declare that they feel a greater pressure than usual to be productive, on account of the pandemic and changes to the way online learning affected their schedule. Additionally, most of them point to themselves as the source of that pressure (*n* = 648, 58.9%). The results indicate that the higher the perceived pressure to be productive, the greater the GHQ total score (*p* = 0.03). In practice, the perceived pressure translated into a success in plan execution and satisfaction with one’s productivity in only 19% (*n*= 183) of the respondents, which in turn led to feelings of guilt and frustration in close to 80% of them (*n* = 880, 79.8%). This observation is also reflected in the levels of psychopathological symptoms. Much higher GHQ scores were found in the group that was not able to succeed in their plans or satisfied with their productivity, compared to the group that was (mean 44.2 vs. 27.08, *p* < 0.0001). Moreover 89% (*n* = 591) of the former group was above the established 24-point cut-off for psychological distress. Similarly, as observed before, successful planning and satisfaction with one’s productivity also significantly influenced the level of insomnia and PTSD symptoms.

#### 3.1.2. Self-Care, Social and Romantic Relationships

As a result of the pandemic, almost a third of the studied group (*n* = 269, 27%) declared they have neglected personal hygiene, 36% (*n* = 349) have neglected tidiness, 43% (*n* = 425) meals, 62% (*n* = 612) activities, 53% (*n* = 517) their interests, 25% (*n* = 234) their family and 53% (*n* = 519) have neglected their friends. All of the aforementioned groups had a significantly higher GHQ total score. In all cases, close to 90% of the respondents who indicated having neglected one of the above areas score above the 24-point cut-off that indicate the presence of relevant psychopathological symptoms. All of the described areas also were significant associated factors of symptoms of insomnia and higher scores on the IES-R. A considerable percentage of the respondents 16% (*n* = 158) stated that their relationship deteriorated or ended during the pandemic, which corresponded with significantly higher GHQ scores in this group, compared to those who did not have that experience. This group also had the highest percentage of scores above cut-off for psychological distress (92%). A similar effect was observed for insomnia and PTSD symptoms.

#### 3.1.3. Food, Sleep, Sex and Physical Activity

Food| The largest group in the studied population 35% (*n* = 340) indicated an increase in food consumption due to the pandemic, followed by no declared change in 23% (*n* = 227) and finally a decrease, observed by 22% (*n* = 219) of the respondents. Furthermore, an equal number of respondents 10% (*n* = 97) declared having taken up a new diet during the pandemic, as have discarded the diet they were on before. The dietary habits were significantly related to the GHQ scores, with highest total scores in the group who gave up their previous diet on account of the pandemic, followed by people who eat less than they did before the pandemic, more than they did before, have started a new diet and the lowest scores in the group that made no change to their food intake. Post hoc analyses point to significant differences between no change in food intake and all of the other responses (more, less, new diet, no diet) as well as giving up a previously maintained diet and eating more and being on a new diet. Changes in food intake were also significantly related to insomnia and PTSD symptoms, similarly with high scores for giving up a previous diet (post hoc significance for no change vs. the remaining groups).

Sleep| The majority of the studied population, 79% (*n* = 630), declare having noticed a change in their sleeping patterns during the pandemic, followed by those who have noticed no change in the amount of time they sleep 21% (*n* = 201). The respondents indicating changes in sleep scored significantly higher on GHQ, than the group who experienced no changes in sleep. The same pattern can be observed with insomnia and PTSD symptom scores.

Sex| A small number 6.3% (*n* = 62) of the studied group indicate the onset of previously absent sexual dysfunctions (i.e., erectile dysfunctions, loss of sex drive). The highest scores on psychopathological symptoms were observed in the group that indicated an onset in sexual dysfunction where, notably, all of the participants scored above cut-off for psychological distress (*n* = 62, 100%). The same was found for insomnia symptoms, with highest scores for sexual dysfunctions and PTSD symptoms.

Physical Activity| Over half of the studied population (60%, *n* = 587) declare their physical activity level has decreased during the pandemic. The physical activity level corresponded with psychological distress, where the group with lower activity levels exhibited significantly higher GHQ scores. The group that declared a decrease in physical activity also scored significantly higher on insomnia symptoms, but not PTSD symptoms.

#### 3.1.4. Psychiatric and Psychological Treatment, Self-Harm and Substance Use

In the studied sample, 161 participants (16%) reported having been in psychiatric treatment at any point in their life, most of them reported being treated for depression and anxiety disorders, followed by eating disorders, OCD, personality disorders and bipolar disorder, out of those, however only 89 (9.1%) report undertaking psychopharmacology treatment. Another 350 (36%) participants confirmed having contact with a psychologist or receiving psychotherapy. The groups receiving psychiatric treatment, psychotherapy or psychiatric medication in the past scored significantly higher on the GHQ as well as ISI and IES-R.

Self-harm| Although the majority of the studied group reported no previous history or current self-harm (*n* = 721, 73.4%), among those who do 16% (*n* = 155) observed no change in frequency, 8.8% (*n* = 86) report an increase and only 1.8% (*n* = 18) a decrease in self-harm behaviors. The results indicate significant differences in GHQ scores between respondents who never engaged in self-harming behaviors and those who did, as well as between those who noted an increase in the frequency of self-harm and all of the other groups with an increase in GHQ total score accompanying an increase in the frequency of self-harming behaviors. The group of respondents reporting an increase in self-harm almost entirely scored above the cut-off for psychological distress (98%, *n* = 84). An increase in self harm also significantly corresponds to insomnia and PTSD symptoms.

##### Substance Use

Alcohol| Only 18% (*n* = 178) of the studied group declared that they have increased their alcohol consumption during the pandemic, compared to 43% (*n* = 420) who report having decreased their consumption in that time. Nevertheless, the results remain related to psychological distress, where the former group score significantly higher on the GHQ total score than the latter and exhibit the highest percentage of scores above cut-off (88%). Increased alcohol consumption is also significantly related to symptoms of insomnia and PTSD. The lowest levels of psychopathological symptoms are observed in the group who declare that they have not changed the amount of alcohol they consume on account of the pandemic; this group also exhibits the smallest percentage of results above the cut-off for psychological distress (75%), compared to the ones who reported to have increased (88%), decreased (78%) or never consumed alcohol at all (79%). The post hoc analyses revealed that it was an increase in alcohol consumption that was statistically significant in relation to all of the other groups (i.e., decrease, same as usual, never used). This was true for all of the aforementioned variables (GHQ, ISI, IES-R). For the purpose of reporting, the responses in the group were dichotomized into ‘increase in alcohol consumption’ and ‘no increase in alcohol consumption’ yielding similar results (for details see Table 2).

Tobacco| An increase in tobacco consumption was reported by 11% (*n* = 111) of the studied population and a decrease by 16% (*n* = 152). The group that noted an increase in tobacco use was not significantly higher on psychological distress. It was, however, significantly higher in insomnia and PTSD symptoms, with highest scores for people who increased consumption during the pandemic and lowest scores, similarly as with alcohol, for those who made no change. The post-hoc analyses similarly point to an increase in consumption as the significant variable. For the purpose of reporting, the responses in the group were dichotomized into ‘increase in tobacco consumption’ and ‘no increase in tobacco consumption’ yielding similar results (for details see Table 2).

Other substances| The survey also included questions related to marijuana, opiates, psychedelics, stimulants and “other”, with no statistically significant results across all of the variables.

#### 3.1.5. Multiple Linear Regression

A multiple linear regression was performed to predict GHQ-28, ISI as well as IES-R scores as dependent variables, in relation to age, sex and the lifestyle questionnaire items. The results of the regression analysis suggest most of the independent variables were predictors of the GHQ-28 scores, but not of the remaining questionnaires (IES-R, ISI). Neglecting interest has the strongest determination on an increase in GHQ scores (β = 0.4943, *p* < 0.001), directly followed by the onset of sexual dysfunction (β = 0.4907, *p* < 0.001), both of the independent variables also significantly reflected on ISI and IES-R scores in the same manner. The second highest, significant negative beta-coefficients for GHQ-28 were noted for the ability to execute plans and productivity as well as self-harm frequency but only for the group that never self-harmed (β = −0.3936, *p* < 0.001; β = −0.369, *p* < 0.001, respectively). Apart from that, the ability to execute plans and productivity were not significant influence determinants of any of the other variables, and self-harm frequency for the group that never self-harmed has only been able to predict the IES-R score (β = −0.2737, *p* < 0.001). Other significant predictors for the GHQ-28 with a beta-value > 2 included the ability to maintain an everyday routine, perceived pressure on productivity, neglecting tidiness, neglecting family, an increase in alcohol consumption and an increase in self-harm frequency, several variables also predicted GHQ-scores with a beta-value < 1. Other determinants of ISI scores included female sex, neglecting tidiness, neglecting meals, neglecting family, a change in food intake and an increase in self-harm. Other predictors of IES-R scores included female sex, perceived pressure on productivity, neglecting tidiness, neglecting family, an increase in alcohol consumption, as well as an end or deterioration of a relationship. Detailed results can be found in Table 3 below.

## 4. Discussion

The presented findings paint a picture of profound changes in lifestyle and everyday functioning experienced by college students in Poland during the pandemic.

### 4.1. Maintaining an Everyday Routine

A considerable percentage of respondents declared having neglected a vast portion of their everyday life including hygiene, tidiness, meals, activities, interests, family and friends, all of which were related to higher psychological distress. Another less pronounced, however significant change affected romantic relationships which have experienced a deterioration or ended for 16% of the studied population significantly affecting their psychological wellbeing. Similarly in the study by Li et al. [29] of 967 participants, 31% reported a deterioration in their romantic relationship during the pandemic. In accordance with our results, that suggest that the ability to maintain a daily routine was significantly related to lower psychological distress, regularizing and maintaining daily routines has been previously mentioned as a protective factor during the COVID-19 pandemic [30]. A simple Google search on the question on how to deal with the pandemic, will yield numerous results from lifestyle websites with advice on coping, that mention keeping a daily routine as an important factor to minimize stress and decrease risk for adverse psychological outcomes. This recommendation is supported by research, where maintaining a daily routine as a protective factor is addressed in several studies, e.g., on post-traumatic stress during natural disasters, e.g., [31] or populations living in high-stress environments, like forced immigrants [32]. Another interesting, however concerning finding is, that although the majority of our respondents endorsed the belief that a daily routine is an important factor influencing the way they feel, only over a third were in fact able to maintain it during the pandemic. This suggests that in this case the belief that something is beneficial was not sufficient to translate into behavior, however, as the question on why routine was not kept has not been further explored in our study, no ready explanation for this contradiction is available. Possible reasons could include the vast changes that affect the individual’s environment and living conditions, that may limit or entirely prevent either physical access to previous routines (i.e., the closing of schools, libraries, gyms, restaurants, malls and public areas) or their individual availability (loss of income due to restrictions on businesses, loss of need for apartments and moving back home due to online learning), with little or no substitutes available. The aforementioned observation also points to the possibly limited efficacy of informational campaigns focusing on advice without addressing the apparent obstacles in adherence to the given recommendations.

### 4.2. Food, Sleep, Sexual Activity and Physical Activity

In addition to a change in daily routines, habits and living environment, our findings suggest a significant impact of changes to physiological routines on the mental health of university students during the pandemic.

The results suggest the majority of the studied population experienced a change in sleeping patterns on account of the pandemic with over 80% of this group scoring above psychopathological distress, suggesting sleep disturbances to be closely linked to the subjective worsening of mental health. The high prevalence of sleep disturbances in the studied group could be linked to the fact that university students generally experience more problems with sleep [33]. However, the questionnaire design referring to changes that occurred after the onset of the pandemic, rather than generally experienced issues, should have eliminated or at least diminished the impact of that explanation on the results. Several previous studies have associated sleep disturbances during the pandemic with experiencing anxiety and stress [34,35]. Interestingly, according to our research, apart from female sex all of the variables that had a significant impact on ISI-scores were neglecting important areas of life (tidiness, meals, interests, family), increase in self-harming behaviors and an onset of sexual dysfunction. All of those can be seen as symptoms of depression, where sleeping problems also appear as a prominent feature.

A similar trend was found for sexual disfunctions with the entire group (100%) who reported experiencing problems of this nature scored above the cut-off for psychological distress. Previous studies have reported issues around sexual and reproductive health amidst the pandemic [29]. Duran et al. [36] investigated whether there has been an increase in the number of patients presenting with sexual and reproductive health issues during the pandemic and found an increase in andrological diagnoses, male reproductive or sexual health problems and erectile dysfunction. The authors conclude that although the etiology is multifactorial, psychological factors seem to play a vital role. Our conclusion of an increased burden on mental health in the group who experienced sexual dysfunctions is in line with the research conducted by Mollaioli et al. [37], who demonstrated that a lack of sexual activity during the COVID-19 pandemic significantly increased the risk of anxiety and depression in the studied population, concluding that sex has a protective effect on mental health. The results of the multiple linear regression also indicate the onset of sexual dysfunction to be a significant predictor for higher scores on all of the measured variables, including psychological distress, sleeping problems and PTSD symptoms (i.e., GHQ-28, ISI and IES-R).

Some early studies on eating behaviors amidst the COVID-19 crisis have already reported changes in food intake as a result of the pandemic [38,39,40,41,42], with the directions of the change varying from a decrease, through no change and finally an increase in food intake. The lowest scores were obtained in the group who made no change to their eating habits, however, similarly to previous research a majority of our respondents reported a change in eating habits since the onset of the pandemic. The respondents who reported eating less and scored second highest in psychological distress, insomnia and PTSD symptoms, could be considered in two ways. Although stress is commonly associated with emotional overeating, research suggests that the effect of stress on food intake can be expressed in both directions i.e., by either an increase or decrease in food consumption [43,44], with some studies suggesting a 50/50 distribution of both tendencies in the studied population [43]. A pandemic-related reduction in food intake, could therefore be considered a response to stress. A second approach to the findings stems from a psychopathological perspective, where patients suffering from eating disorders (ED) (e.g., anorexia nervosa) have been significantly impacted and constitute an at-risk group for adverse consequences of the COVID-19 pandemic and lockdown [45,46]. Although the effects of the pandemic on the ED population still remain unknown, an increase in eating disordered symptomatology (e.g., dietary restrictions, binge episodes, emotional eating) has been reported as an effect of the pandemic in ED clinical populations [47]. On the other end of the eating spectrum, the vast majority of the studied group (35%) reported an increase in food consumption, which seems to be in line with previous studies, e.g., by Buckland et al. [38] who reported 48% of the participants experiencing an increase in food intake due to the COVID-19 pandemic. The results of an automated questionnaire, conducted on an unspecific population by a bariatric multidisciplinary team of Cherikh et al. [48], suggest that 37–43% of the studied group managed stress, feelings of emptiness and boredom with food consumption. The aforementioned study also found a significant positive correlation of those variables with lower physical activity, suggesting that individuals who engage in emotional eating simultaneously exhibit low levels physical activity, which naturally poses a significant threat as far as weight gain is concerned. A decrease in physical activity has previously been reported as a consequence of the pandemic [49] and is reflected in the results of our study where it also significantly corresponded with psychopathological distress in line with a study conducted by Ellingson et al. [50] on a population of American adults, who concluded, that large amounts of sedentary time “are associated with poor mood, stress, and sleep”.

### 4.3. Psychiatric and Psychological Treatment, Self-Harm and Substance Use

The results of our study indicate that having received psychiatric treatment, psychotherapy or psychiatric medication was significantly related to higher psychopathological distress, insomnia and PTSD symptoms. Moreover, 16% of participants reported a history of self-harm, which constitutes quite a large number in comparison with a similar survey focusing on self-harm, conducted during the pandemic by Iob et al. [51] where only around 5% engaged in self-harming behaviors. The higher prevalence of self-harming behaviors in our sample could be a result of the specific studied demographic. Our sample consisted exclusively of young adults and was predominantly female (75%), both of which have been found to be risk factors for self-harm [52]. The results of the multiple linear regression analysis also indicate no history of self-harming behaviors to be a predictor of lower psychopathological distress as well as post-traumatic symptoms.

Our findings suggest that, although only a small percentage of the studied population reported an increase in alcohol and tobacco use, both were significantly related to a decrease in psychological wellbeing (i.e., psychopathological symptoms, insomnia and PTSD symptoms). A study by Lechner et al. [53] conducted among college students at the onset of the pandemic and campus closure, similarly reported an increase in alcohol consumption with an additional finding that those students who reported depression or anxiety demonstrated a higher increase in alcohol consumption. An explanation for why only 18% of the studied group reported an increase in consumption of alcoholic beverages, compared to 43% who have decreased their alcohol intake could be related to habits of university students in Poland. Most people attending university in Poland are not based on campus, i.e., in dormitories but rent flats, either alone or with roommates that they are not necessarily well acquainted with, especially during the initial years of college, which in our study constitutes almost half of the group. Moreover, the ones that do live in dormitories were asked to vacate their rooms soon after the lock-down started. The closing of social gathering sites and events that accompanied the closing of universities and onset of online classes could have significantly reduced drinking occasions. It therefore limited the amount of consumed alcohol, as opposed to environments where students could still gather in their communities. This hypothesis is also supported by the aforementioned study, where the authors have found that an increase in the consumption of alcoholic drinks was accompanied by an increase of drinking occasions overall, which at that time in Poland was unlikely.

### 4.4. Life-Skills

The described results identified protective and risk factors in relation to mental wellbeing and psychopathological symptoms could also be understood in the context of life skills. The World Health Organization (WHO) defines life skills as “abilities for adaptive and positive behavior, that enable individuals to deal effectively with the demands and challenges of everyday life.” In this context, those respondents who were able to deal more effectively with the changing circumstances of the pandemic also reported better mental health. Moreover, an interesting observation is that the lowest psychopathological and insomnia scores were observed by those who made no change in both cases: alcohol and tobacco. These could be understood, in the context of the previously discussed findings, where the lowest scores were also observed for the group who made no change to their eating habits as well as those who were best able to maintain a daily routine. It therefore stands to reason that making no changes to the areas where no change is immediately required, may be a protective factor by itself and the lowest psychopathological scores were obtained by that part of the group who was possibly better equipped to deal with stressful situations in the first place. According to WHO, among others, life-skills include abilities such as problem solving, effective communication skills, decision-making, creative thinking, interpersonal relationship skills and coping with stress and emotions. If a reasonable level of all of the above has been acquired in the time before early adulthood, crises and environmental changes, such as the pandemic, could be handled better in terms of psychological wellbeing. In our study, maladaptive coping strategies, such as emotional eating, alcohol as well as self-harm, were directly linked to higher levels of psychological distress. The same was true for interpersonal skills, when considering neglecting family and friends as well as the deterioration of relationships.

The UNICEF definition of life skills “a behavior change or behavior development approach designed to address a balance of three areas: knowledge, attitude and skills” puts more emphasis on the components that constitute a behavior change (or behavior). This emphasis is especially important in the context of our research, where, for instance, despite an abundance of informational campaigns pointing to nutrition, physical exercise and routine as important factors for maintaining psychological and physical wellbeing during the pandemic, the results suggest these were not implemented in practice. The same is true when considering the majority of the studied group agreed a daily routine is an important factor influencing the way they feel, only over a third were able to maintain it during the pandemic. These finding underline the problem that simply conveying knowledge is possibly not enough to elicit a behavioral response and additional criteria like attitude and skills play an important role. Although all of the abovementioned factors influencing the choice of behavioral strategies and the adherence to informational campaigns assume the skills to do so would have to be present beforehand, i.e., underline the necessity of life-skills training programs before crises occur, they also point to the areas that should be targeted to improve psychological wellbeing in times of elevated stress and do not have to be treated exclusively in hindsight. Even though the majority of the research on life-skills training programs focuses on younger age groups [54], studies have also pointed to the efficacy of targeted life-skills interventions in college students and adults [55,56,57].

### 4.5. Generalizability of the Results

As the study was carried out on a group of university students in Poland, the generalizability of the results may be limited by specific circumstances relating to the willingness to participate, group characteristics as well as the cultural environment. Since the survey was voluntary and the motivation to participate was not addressed, no data on reasons for participation is available. Due to the distribution of the group, where most students who decided to participate were in their first or second academic year (46%), it stands to reason those students had more reasons to take part in the study than more advanced students, who participated in much lower numbers (16%). As previously discussed, this could be related to the fact that the impact of the pandemic, and therefore the experienced psychological consequences, may be much higher for students who have just started their academic career, compared to already settled in and assimilated students from higher years. The motivation to participate could therefore stem from a higher need for an outlet for the negative emotions and stress created by the pandemic. Due to their academic status and young age, university students in Poland are typically reliant on their parents for financial support and sometimes keep low-paid part-time jobs, and access to financial help from university is low. In our study this is reflected by 74% of the group being unemployed and 83% supported by their parents. Smaller financial resources may be a factor preventing better adaptation as far as mobility, food choices and exercise options are concerned (paid gyms, food and gas prices) as well as an additional source of stress. Apart from cultural and economic factors, as mentioned previously, college students are more vulnerable to mental health problems, compared to the rest of the population [5] at baseline. This is also supported by findings from a recent Chinese study, conducted by Wang et al. during the coronavirus pandemic, where student status was associated with a larger psychological impact of the pandemic as well as higher stress, anxiety, and depression symptoms [58]. In the same study, another factor associated with poorer mental health during the pandemic was the female gender [58] and a similar finding was also reported by a recent study conducted in Poland [3]. As the majority of the studied group was female (75%) this factor could have significantly influenced the results.

## 5. Conclusions

The presented findings paint a picture of profound changes in lifestyle and everyday functioning of university students in Poland during the SARS-CoV-2 pandemic, underlying the need for additional support in those populations. Based on the obtained results we were able to establish a set of data that, in the context of previous research, can be interpreted as a list of protective and risk factors. Protective factors include maintaining a daily routine, staying physically active, following a usual eating pattern and taking care of sleep hygiene. In summary, making no changes, where no changes are needed (e.g., due to the necessity to adapt to a change in environment) seems to be a protective factor by itself. Based on the results, risk factors for poorer mental health include giving up a daily routine, including neglecting meals, tidiness, hygiene as well as social relationships, changes in food intake, sleeping schedule and a decrease in physical activity and the onset of sexual dysfunctions. Previous psychiatric treatment, a history and increase in self-harm as well as an increase in alcohol and tobacco consumption have also been found to be detrimental to mental health. The results imply that targeted support interventions addressing life-skills and improving coping in stressful situations early on may decrease the likelihood of adverse psychological outcomes in young adults later. To date several studies have addressed this issue in a practical manner in reference to the pandemic, e.g., progressive muscle relaxation has been found to improve anxiety and sleep in patients with COVID-19 [59]. Similarly, peer-education interventions have been found to improve anxiety, depression and sleep quality in a study of adolescents in China [60]. Physical activity and nutritional guidelines that may be used for informational support interventions have also been published [61,62]. Programs that directly address life-skills as well as training materials and recommendations are readily available online as well as through the WHO website.

## 6. Limitations

The major limitations of the study are simultaneously limitations associated with the epidemiological situation at the time of data collection and its implications to research methodology. Firstly, despite the use of CAWI methodology, some limitations due to the online format of the questionnaire remain. Despite the fact that research indicates that the demographic most likely to take part in internet surveys are younger people, this aspect of the selection bias may be less apparent in our study as it was aimed exclusively at college students. There may, however, be an underrepresentation of an older demographic of students. As the access to the study was partially distributed by the help of universities across Poland, a selection bias concerning which universities decided to publish the study is applicable in the case of this study. This was partially corrected by aiming at students’ organizations and social media, to avoid distributing the survey via university websites alone. Due to the emphasis on absolute anonymity, the collection of e-mail addresses for follow-up was conducted in a voluntary manner and hence, no follow-up data could be obtained. Due to the sudden onset and global character of the pandemic, no control group of college students could have been obtained. After the statistical analysis of the results, it became apparent that several of the results need further exploration, i.e., the reasons for inability to maintain a daily routine, reasons for increase/decrease in alcohol, tobacco consumption, change in eating habits. We hope to address these research questions in separate studies. Due to the lack of a control group, the results should be interpreted with caution. The study design as well as the measurements used rely on subjective self-reports, no objective measures of the studied variables (e.g., physical activity, food intake, sleep patterns and alcohol and tobacco consumption) were included. The results should therefore be treated as a reflection of the students’ self-experiences during the pandemic rather than an objective description of measurable behavioral changes.

## Figures and Tables

**Table 1:** Demographic characteristics of the study sample, *n* = 980.

Characteristic	*n*	%
**Demographic**		
**Sex**		
Male	247	25
Female	733	75
**Age**	Mean 22.24, SD (2.46), median 22.00 (21.00, 23.00)	
**Place of residence**		
<100 thousand	463	47
>100 thousand	518	53
**Employment**		
Unemployed	728	74
Employed	252	26
**Source of income**		
Partner	25	2.6
Family	817	83
Self-supportive	138	14
**Education**		
**Field of study**		
Medical	305	31
Technical	262	27
Other	413	42
**Full-/part-time**		
Full-time	908	93
Part-time	71	7.3
**Year**		
1st and 2nd	447	46
3rd or 4th	368	38
5th or 6th	152	16
Other	13	1.3
**Covid-19 Exposure**		
**Infected**		
Yes	1	0.1
No	979	99.9
**Quarantined**		
No	945	96
Yes	35	3.6
**Quarantined family member**		
Yes	70	7.1
No	910	93
**Death in family**		
No	970	99
Yes	10	1.0

**Table 2:** Differences in GHQ-28, ISI, IES-R^5^ scores between groups based on their questionnaire responses.

Questionnaire Item	Response		GHQ		ISI		IES-R	
			Mean (SD)	Effect Size ^2^, *p* ^3^	Mean (SD)	Effect Size ^2^, *p* ^3^	Mean (SD)	Effect Size ^2^, *p* ^3^
**Ability to maintain everyday routine**	1	No	43.21 (16.30)	0.2795 ***(r)	10.35 (5.84)	0.171140 ***(r)	37.82 (18.07)	0.12208 **(r)
	2	Yes	32.93 (17.31)		8.26 (5.76)		32.94 (19.07)	
**Perceived pressure on productivity**	1	No	35.80 (18.08)	0.01129 *	ns ^1^	--	ns	--
	2	Yes	40.78 (17.00)					
**Ability to execute plans and productivity ^5^**	1	No	35.12 (15.63)	0.152 ***	10.43 (5.83)	0.0385 ***	37.98 (17.87)	0.02098 ***
	2	Yes	27.08 (15.22)		7.62 (5.57)		30.74 (18.89)	
**Neglecting personal hygiene**	1	No	36.77 (16.68)	0.07311 ***	9.14 (5.87)	0.02124 ***	34.54 (18.22)	0.02301 ***
	2	Yes	47.29 (16.66)		11.04 (5.78)		40.99 (18.53)	
**Neglecting tidiness**	1	No	35.55 (16.52)	0.09934 ***	8.75 (5.79)	0.04485***	33.41 (18.32)	0.0347***
	2	Yes	46.98 (16.50)		11.36 (5.79)		41.08 (17.97)	
**Neglecting meals**	1	No	34.61 (15.73)	0.10821 ***	8.20 (5.54)	0.07725 ***	33.49 (18.01)	0.0216 ***
	2	Yes	46.50 (16.81)		11.54 (5.84)		39.58 (18.88)	
**Neglecting activities**	1	No	34.45 (16.95)	0.05046 ***	8.65 (5.71)	0.01758 ***	ns	--
	2	Yes	43.09 (16.79)		10.32 (5.89)			
**Neglecting interests**	1	No	30.76 (15.58)	0.21141 ***	7.54 (5.41)	0.10569 ***	30.75 (18.33)	0.06144 ***
	2	Yes	46.95 (15.48)		11.40 (5.79)		40.63 (17.72)	
**Neglecting family**	1	No	36.96 (16.53)	0.07136 ***	9.02 (5.69)	0.02662 ***	34.36 (18.32)	0.02267 ***
	2	Yes	46.47 (17.65)		11.37 (5.69)		41.03 (18.98)	
**Neglecting friends**	1	No	34.76 (16.17)	0.06108 ***	8.57 (5.49)	0.02275 ***	33.15 (18.21)	0.01776 **
	2	Yes	43.64 (16.94)		10.56 (6.10)		38.46 (18.66)	
**Increase in alcohol consumption, compared to pre-pandemic**	1	No	38.36 (16.88)	0.1915 ***(r)	9.29 (5.77)	0.13911 ***(r)	34.73 (17.99)	0.16844***(r)
	2	Yes	47.05 (17.42)		11.47 (6.11)		43.20 (19.36)	
**Increase in tobacco consumption, compared to pre-pandemic**	1	No	39.2 (17.27)	0.11897 **(r)	9.31 (5.83)	0.18202 ***(r)	35.42 (18.43)	0.12765 **(r)
	2	Yes	45.7 (16.47)		12.68 (5.59)		42.86 (18.03)	
**Relationship with partner deteriorated or ended**	1	No	38.50 (17.23)	0.18757 ***(r)	9.33 (5.84)	0.14002 ***(r)	35.07 (18.28)	0.13917 ***(r)
	2	Yes	47.41 (15.72)		11.56 (5.82)		42.48 (18.61)	
**Onset of sexual dysfunctions**	1	No	38.82 (16.94)	0.23802 ***(r)	9.39 (5.81)	0.19548 ***(r)	35.41 (18.39)	0.17012 ***(r)
	2	Yes	56.48 (13.83)		14.15 (5.37)		48.90 (15.83)	
**Change in food intake, compared to pre-pandemic**	1	No change	32.79 (16.42)	0.0621 ***	7.48 (5.09)	0.04879 ***	31.41 (18.22)	0.01659 *
	2	I eat less	43.92 (17.41)		11.04 (6.02)		38.69 (18.44)	
	3	I eat more	40.34 (16.99)		9.82 (5.97)		36.87 (18.83)	
	4	Stopped previous diet	46.64 (15.87)		11.26 (5.45)		39.00 (17.33)	
	5	Started new diet	39.59 (15.95)		9.77 (6.09)		37.33 (17.74)	
**Post hoc ^4^:**		1.2 ***; 1.3 ***; 1.4 ***; 1.5 **			1.2 ***; 1.3 ***; 1.4 ***; 1.5 *		1.2 ***; 1.3 *; 1.4 *	
**Change in sleeping patterns, compared to pre-pandemic**	1	No	35.71 (16.91)	0.12135 ** (r)	8.50 (5.65)	0.10082 ** (r)	ns	--
	2	Yes	41.01 (17.24)		9.99 (5.92)		ns	
**Decrease in physical activity, compared to pre-pandemic**	1	No	35.58 (17.50)	0.20422 ***(r)	8.69 (5.75)	0.13793 ***(r)	ns	--
	2	Yes	42.86 (16.55)		10.35 (5.90)			
**Self-harm (frequency), compared to pre-pandemic**	1	No change	46.13 (15.90)	0.11034 ***	10.16 (6.07)	0.03368 ***	40.69 (17.15)	0.04554 ***
	2	Never self-harmed	36.67 (16.47)		9.12 (5.68)		33.90 (18.50)	
	3	Increased	42.17 (13.73)		11.28 (5.54)		46.39 (15.67)	
	4	Decreased	55.72 (15.50)		13.23 (6.10)		46.01 (16.50)	
**Post hoc ^4^:**		1.2 ***; 1.3 ***; 2.3 ***; 2.4 **			1.3 *; 2.3 ****		1.2 ***; 1.4 *; 1.3 ***	

^1^ Non-significant differences were omitted or marked as “ns”. ^2^ The non-marked effect sizes represent η^2^, the effect sizes in Wilcoxon r are marked as “(r)” and range between <0.1 insignificant, <0.3 small, <0.5 moderate, >0.5 large effect. ^3^ P-value: <0.05 *, ≤0.01 **, ≤0.001 ***. ^4^ Post-hoc analysis for multiple responses as numbered with p. adj. significance (*). ^5^ Some of the questions included an “I don’t know” answer, these were neither included nor interpreted in the results.

**Table 3:** Results of the multiple linear regression analysis for questionnaire responses and GHQ-28, ISI and IES-R.

*Predictors*	GHQ-28			ISI			IES-R		
	*Beta*	*Std Error*	*p*	*Beta*	*Std Error*	*p*	*Beta*	*Std Error*	*p*
**(Intercept)**	−0.9670	0.1202	<0.001	−0.9843	0.1426	0.017	−0.7479	0.1469	<0.001
**Age**	−0.0867	0.0249	0.001	−0.0070	0.0295	0.813	−0.0306	0.0304	0.315
**Sex (Female)**	0.1430	0.0563	0.011	0.1858	0.0668	0.006	0.1841	0.0688	0.008
**Ability to maintain everyday routine**	−0.2299	0.0546	<0.001	−0.1091	0.0648	0.093	−0.0491	0.0667	0.461
**Perceived pressure on productivity**	0.2119	0.0649	0.001	0.1382	0.0770	0.073	0.1870	0.0793	0.018
**Ability to execute plans and productivity**	−0.3936	0.0698	<0.001	−0.0361	0.0842	0.668	0.0026	0.0867	0.976
**Neglecting personal hygiene**	0.1977	0.0584	0.001	0.0537	0.0693	0.439	0.1106	0.0714	0.122
**Neglecting tidiness**	0.2252	0.0558	<0.001	0.1334	0.0663	0.044	0.1824	0.0682	0.008
**Neglecting meals**	0.1734	0.0559	0.002	0.2493	0.0663	<0.001	−0.0008	0.0683	0.990
**Neglecting activities**	−0.1081	0.0743	0.146	−0.1627	0.0882	0.065	−0.1053	0.0908	0.246
**Neglecting interests**	0.4943	0.0572	<0.001	0.3582	0.0679	<0.001	0.2929	0.0699	<0.001
**Neglecting family**	0.2014	0.0611	0.001	0.1447	0.0725	0.046	0.1493	0.0747	0.046
**Neglecting friends**	0.1872	0.0543	0.001	0.1180	0.0644	0.067	0.0860	0.0664	0.195
**Increase in alcohol consumption, compared to pre-pandemic**	0.2032	0.0655	0.002	0.0914	0.0777	0.240	0.2480	0.0801	0.002
**Increase in tobacco consumption, compared to pre-pandemic**	0.0110	0.0784	0.888	0.3359	0.0930	<0.001	0.1681	0.0958	0.080
**Relationship with partner deteriorated or ended**	0.1852	0.0683	0.007	0.1118	0.0811	0.168	0.1953	0.0835	0.020
**Onset of sexual dysfunctions**	0.4907	0.1037	<0.001	0.4070	0.1230	0.001	0.3702	0.1267	0.004
**Change in food intake, compared to pre-pandemic**	0.1900	0.0595	0.001	0.2114	0.0706	0.003	0.1312	0.0727	0.071
**Change in sleeping patterns, compared to pre-pandemic**	0.0592	0.0602	0.326	0.0725	0.0714	0.310	0.1237	0.0735	0.093
**Decrease in physical activity, compared to pre-pandemic**	0.1810	0.0692	0.009	0.1391	0.0822	0.091	0.0990	0.0846	0.242
**Self-harm (frequency), compared to pre-pandemic (never)**	−0.3690	0.0675	<0.001	−0.0570	0.0801	0.477	−0.2737	0.0825	0.001
**Self-harm (frequency), compared to pre-pandemic (less)**	0.0552	0.1883	0.769	0.3794	0.2234	0.090	0.3983	0.2301	0.084
**Self-harm (frequency), compared to pre-pandemic (more)**	0.2467	0.1025	0.016	0.2922	0.1217	0.017	0.0781	0.1253	0.533

## Data Availability

Not applicable.
